# Bibliographical Notices

**Published:** 1837-01-01

**Authors:** 


### Periscope;

OR,

### CIRCUMSPECTIVE REVIEW.

" Ore trahit quodcunque potest, atque addit acervo."

### Notices of some New Works.

Retrospective Address, delivered at the Fourth Anniversary Meeting of the Provincial Medical and Surgical Association, held at Manchester, July 21st, 1836. By John Green Crosse, Esq. F.R.S. &c.

This Address is contained in the volume of the Provincial Transactions, published at the same time as the present Number of our Journal. Through the kindness of Mr. Crosse, we have been favoured with a glance at his interesting paper, and take the opportunity of offering a slight sketch of it, and of making a few extracts from it.

The object of Mr. Crosse is to exhibit the improvements that have been effected in any of the departments of medicine in the couise of the preceding year. The industry of Mr. Crosse ensures his doing justice to the voluntary task, and he has done justice to it.

It would be pieposterous pursuing the details of an address of this sort. In most instances, it must offer a mere catalogue of papers published or of facts related, and, at all events, many, if not most of both have been already noticed in the pages of this and of the other journals. We shall, therefore chiefly occupy ourselves in picking out a fact or two here and there.

1. *Anatomy.* Mr. Crosse observes with great justice, that the diffusion of anatomical knowledge is remarkable. It is, indeed, surprising what a rapid stride hasbeen made within these veryfew years in the mode of teaching anatomy, and in the amount that is acquired by students. Systems of anatomy that a little while ago were considered excellent, and were received as standard works, are now neglected as superficial or condemned as inaccurate. Teachers who were amongst the most popular of their day, are now deserted, or regarded as the representatives of a system that is virtually extinct; and pupils who formerly would have been regarded as prodigies of anatomical information, would now stand little chance of obtaining a prize in any first-rate London school. These improvements are generally attributable to the spirit of the age—their particular source may be found in the brilliant results that flowed from physiological experiments, in the attention that was naturally directed to anatomy, in the more strict application of geometrical principles to its investigation and description, and, finally, in the competition of the schools themselves.

It is not, then, in the discovery of new facts in anatomy that we shall find much cause for congratulation; but it is in the more philosophical manner of viewing established facts, and in the more precise and scientific mode of teaching them, that our advance is most perceptible. The good that will spring from this is greater than at first sight it may appear. The student does not merely acquire a few facts and principles in the brief but eventful period of his pupillage—his professional mind is then in a great measure formed, and its subsequent bent will materially depend on the impulse given to it. If exact habits of thought are nurtured, if precision of observation, of ideas, and of description is encouraged, we may anticipate the best fruits in after-life from such intellectual education and discipline.

The anatomical discoveries of the year are thus summed up by Mr. Crosse.

Drs. Breschet and Roussel, trusting to microscopical observations, have minutely described the anatomical appearances of the skin, and satisfactorily demonstrated the sudorific exhalent ducts; the inhalents they have not been able to follow to a termination on the outer surface of the skin, and wish, therefore, that their account of them should be received *salvo errore*. Professor Müller has discovered some remarkable appendices connected with the minute arteries of the *corpus spongiosum* and *corpora cavernosa*, which promise to throw light upon the structure of the blood-vessels in all the erectile tissues.

2. *Medical Statistics of the Army.*

"There is a proceeding which I have not found advocated, and which it would well become a liberal Government to adopt. For nearly twenty years past, through the admirable regulations of the present Director-General of the Army Medical Department, returns have been made of all cases of disease in the British army, whether stationed at home or in any of our Colonies; every fatal case from disease has been inspected, and a detailed account of the morbid appearances, often accompanied by the morbid parts when interesting, has been transmitted to the central office: thus documents of the greatest value, referring to the diseases of all climates, and to a limited and well-known population, have been accumulating, until they are become so voluminous, that no chance remains of their being turned to public use, unless a competent person be appointed and properly paid, for putting them in order.* That such a step should be taken seems due to Sir James M'Gregor, whose assiduous and firm pursuit of a wise system, in the medical department over which he presides, has placed in the hands of Government the materials for an annual publication of statistical information, that could not be promulgated without producing great benefit."

We heartily concur in this suggestion of our author's. But "half a loaf is better than no bread," and if we cannot have the documents in arrear, or rather an elaborate analytical digest of them, we can, at all events, have a half-yearly or a yearly report of those which are to come. None can be more disposed than Sir James M'Gregor, to do the utmost both for his particular service and for medical science. We should conceive that there are many assistant-surgeons or surgeons in the army, whose services the excellent Director-General could command, and who would gladly avail themselves of the opportunity of drawing up, with his supervision and sanction, a periodical report. The journals might be made available for its publication, and the only trouble would be that of collecting, comparing, and digesting the materials.

3. *Retraction of the Tongue.* An infant was labouring under symptoms suddenly arising, and seeming to threaten death by suffocation; the physician, fortunately, discovered that the tongue was retroverted, its apex reaching into the œsophagus; the tongue was readily replaced, with relief to all the symptoms, but the trouble recurred frequently. Mr. Crosse knew one instance, where the tongue could be *swallowed* at will without inconvenience. It was in the case of a lad, who, in the playful period of his recovery from fever, was asked to shew his tongue and presented his open mouth with no tongue visible; as soon as his mouth was shut, he asserted that his tongue was in its right place, proving it to be so: there was such facility in retro-

* "The Government have, I am informed, recently appointed a Commission, composed of three gentlemen, of whom two are medical, to inspect and arrange these documents, with a view, it is hoped, to their being promulgated in a useful form. The arrears being disposed of, an annual statistical return could readily be furnished."

verting the tongue into the pharynx, that he frequently repeated the trick afterwards.

4. *Entozoa in the Human Body.*—Since Mr. Owen first discovered the trichinia spiralis in the muscles, these entozoa have been widely and frequently noticed. In a piece of muscle an inch square possessed by Mr. Crosse, there are between one and two thousand. The *cystercus adiposus*, not uncommon in the fat of swine and some other animals, has also been recently, for the first time, observed in the human subject, by a former pupil of Mr. Crosse's, Mr. Baron, abundantly occupying the cellular intervals of many of the voluntary muscles. Dr. Knox has published an account of Entozoa found beneath the intestinal mucous membrane of the horse.

5. *Homœopathy.* The following are Mr. Crosse's opinions on the extraordinary combination of folly and of humbug, which goes under the name of homœopathy. Setting aside the opinions, the statistical facts he records are not uninteresting. They shew, or seem to shew, that even in Germany this most preposterous foolery is on the decline.

"Enthusiasts as extravagant are met with in theoretical medicine, as in the worst forms of religious superstition; but nothing has, I believe, arisen within a century, surpassing or even equalling what has been dignified with the name of the 'homœopathic system.' This germanic reverie of transcendental nonsense—*cui fumus est pro fundamento*—built upon smoke! would not deserve to occupy the time of the present enlightened assembly, had it not made some stir in the country of its origin, choking the press with visionary treatises and worthless periodicals, and spreading gradually through several parts of Europe, and even of the Western world;* a doctrine inferior to Brownism, curing or pretending to cure as magically as by mesmerism, animal magnetism, and the royal touch; a doctrine at variance with every rule of the inductive philosophy, and of the causation of diseases; a doctrine essentially setting forth that the effect is

* "In Germany nearly twenty books were published upon the homœopathic doctrine, in 1832; in the following year twice as many issued from the press; in 1834 the number amounted (according to Dr. Bluff, who has kindly facilitated my enquiries) to sixty-four; and in 1835 I find *only* thirty-one enumerated. The author of a dense volume on this medical mania (*Die Homöopathie von der practischen Seite beleuchtet, ein Lesebush für Aerzte aller Confessionen, herausgegeben von Dr. F. Lesser*, Berlin, 1835,) occupies about twenty pages in referring to different works upon the subject, and quotes the remark that 'the doctrine has spread like the cholera, and will go round the world!' Dr. Lesser specifies twelve homœopathic or anti-homœopathic journals in 1835, (p. 6); four journals are still forthcoming in Germany, one against the doctrine. Notwithstanding the decision of the Royal Academy of Medicine of France, against the system, I find that seventeen homœopathic treatises issued from the press in that country, in 1835, and a journal devoted to it is regularly published in Paris; another journal appears in the same language at Geneva. Italy also has its homœopathic journal, issuing at Naples; Spain had one, which has been *put at rest* by intestine troubles *without assassination.* It is well known that in Russia, physicians were forbidden to practise according to this new system, in public institutions. Dr. Otto remarks that in Denmark but *one* medical man has published upon 'this foolish *system* of medicine, and one of the bitterest satires upon it;' and he thinks the sound sense and judgment of the faculty and the public will prevent its taking root in Danish soil. Holland remains equally free; whilst the profession in Belgium is strongly imbued with the homœopathic disorder, as is abundantly shewn in their medical journals, and in the reports of their discusgreater as the cause applied is less,† and leaving no rational explanation of a cure in the cases where a cure is effected, otherwise than by regulated diet, and the natural restorative powers of the living system, aided by the imagination of a confiding patient. The enthusiast and the charlatan are always industrious, and in our too conjectural art, when any theory, however vague and preposterous, gets afloat, fancies are soon mixed up with some realities, and in time things actually become combined with the imaginary in endless perplexity; thus support is gained, new combinations are formed, and something useful is occasionally elicited even by the worst and vainest of doctrines. It is, however, but the fruitfulness which is found to arise from digging the soil in search of a supposed hidden treasure that does not exist."

Mr. Crosse proceeds to compare homœopathy with the famous cure of wounds, which is well known as Sir Kenelm Digby's sympathetic salve.—This was applied, not to the wound, but to the weapon, and answered marvellous well. But we need not say more upon the subject, which has been touched on in another part of the present number.

6. *What a dangerous person a mere operator is.* Journalism has its evils. A great one is its power of conferring undue reputation on partizans, and of trumpeting one man at the expense of others. This is bad enough under any circumstances, but if qualities are selected for adulation and for puffing, the possession of which is dangerous and the exercise indiscriminate, the evil in a moral, as well as in a professional point of view, is aggravated.

There are few things more calculated for display than operations performed with coolness and dexterity. They appear to the ignorant the triumph of surgery, and the vulgar stare with astonishment and delight at the manipulations of the scientific butcher. Yet such sleight of hand, if not restrained by the higher qualities of intellect, if not guided by caution, directed by experience, and governed by sound reason, is a curse to the community, and a disgrace to medicine. When a hospital is turned into human shambles, and patients are mutilated that operators may shew off, every man possessed of common sense and common decency reprobates the brutal exhibition.

Amongst the really well-informed members of the medical profession, the taste for great operations has happily declined. They are justly considered the disgrace of science, and its progress has been signally and nobly distinguished by the gradual diminution of them. An operation prevented is really a triumph—an operation absolutely necessary, is a calamity—an operation unnecessarily executed is butchery.

When we reflect how small is the number of cases really requiring capital operations—how great is the number in which science may prevent and ignorance perform them—we shall feel how dangerous that man must be, whose chief merit consists in his handicraft

---

sions. America is not quite clear of the same charge, though not burthened with a specific journal. In England we are still more fortunate; we hear, indeed, of 'homœopathic powders,' curing as wonderfully as the *powder of sympathy;* but we are only arrived, so far as I know, at the third book on *homœopatheia,* and from the pen of an author, whose name stands very low in the alphabet. (August, 1836.)"

† "Hahneman assures us he can cure intermittent fever with millioneth, and syphilis with sextillioneth doses of mercury!—*The Lancet.*—When Lord Bacon spoke of 'commanding so over the medicine that the medicine cannot command over the disease,' he never divined of such a system as this, where 'the active properties of each remedy become more developed in proportion to the minuteness of the dose;' in short, 'homœopathists are *cautioned* against *too minute a subdivision of the dose,* lest it should become so energetic as to give rise to dangerous symptoms!!'—*Lee's Observations on the Medical Institutions of France, Italy, and Germany,* p. 203. 1835."

work—and who, incapable, perhaps, of much reasoning, indifferently skilled in the treatment of diseases, is naturally induced to attach most importance to what he best knows, and to prize that which he is conscious of excelling in. In the infancy of surgery, a mere operator would be admired and consulted—its advance would serve to make him shunned. But we return to Mr. Crosse's remarks.

"A fondness for certain operations, and a degree of over-zeal in seeking for occasions to perform them, are modern traits of professional character, which it is to be hoped, will become obliterated or obscured by the daily improvements in pathology and the diagnosis of diseases. The mere operator ought not to be ranked amongst the best of surgeons, and the kidnapping of such patients as furnish conspicuous cases for an hospital theatre, or for the more private assembly of a *coterie* of young operators, should be discountenanced and denounced by every conscientious cultivator of surgical science. Led by a close investigation of symptoms to the necessity of the undertaking, how much more creditable is it for the surgeon to *paracentize* the chest for empyema, than to *lithotomize!*"

7. *Ligature of both Carotid Arteries.* We may refer to the present Number, for an account of the experiments of Sir Astley Cooper on the ligature of the carotid and vertebral arteries in animals. Man, it appears, will not bear the simultaneous stoppage of the current of blood through both carotids.

"Professor Mott* has tested this question, and a case came under my own observation very recently,† shewing how fatal is the tendency of a ligature applied almost simultaneously to each of these arteries. An approximation has, however, been made towards ascertaining the shortest interval at which the second carotid may be safely tied after a ligature to the first, and it has been safely done at an interval of thirty-eight, seventeen, and even twelve days.‡ Amongst the most striking of these cases, is that related by Professor Kuhl, of Leipsig,§ who, on account of a pulsating aneurismal tumour of the scalp, arising from a wound of the occiput, and extending over nearly the entire surface of the head, attended by frequent hæmorrhages, first placed a ligature on the left common carotid; the procedure only partially subduing the disease, and frequent hæmorrhages from the affected portion of the scalp still occurring and threatening life, a ligature was put upon the right common carotid after 27 days; this was followed by convulsions, but after a train of very troublesome symptoms, the patient recovered and was cured of his disease. It is worthy to be noticed

* "Both carotids having been tied nearly simultaneously, with the view of arresting the growth of an enormous tumour in the situation of the parotid gland, the patient survived about twenty-four hours."

† "An attempt having been made to remove a tumour of small size, implicating the right parotid gland, the operator on this sad occasion wounded a large artery deeply situated, anterior to the mastoid process; a ligature was put upon the right common carotid, but without any good effect; the hæmorrhage continuing, I was called on the emergency; tying the left common carotid was suggested, but even *pressure upon it,* so as to interrupt the circulation, *produced insensibility and convulsion,* and seemed to threaten extinction of life; *the experiment* was twice made with the same effects; by a dip of the needle I after some time inserted a ligature which stopped the bleeding vessel. The respect I entertain for professional decorum precluded my again seeing the patient, who lost his life by the undertaking which he was persuaded to believe necessary to save it."

‡ Dr. Mussey in America.—See "*Cyclopædia of Anat. and Phys. by Dr. Todd,*" article "*Carotid Artery.*"

§ The case is related in *Radius and Clarus' Beiträge zur practischen Heilkunde;* also at length in the *London Medical Gazette,* vol. XVI. p. 816.

that in this, and also in other like cases, some days after both carotids had been tied, heaviness and throbbing in the head have occurred, requiring free venæsection, the sensorium being unable to bear the impetus of the returning circulation after having for a time received so scanty a supply of blood."

8. *Ligature of the Gluteal Artery.* This has been lately performed effectually. It is the fourth time that the ligature of the vessel has been recorded.

9. *Operations on Dilated Veins.* Mr. Crosse notices several operations that have been proposed or performed for varices and for varicocele. We dedicated a short article to M. Breschet's mode of treating varicocele. We confess that we feel some degree of repugnance to these operations on the veins. The principles on which they are most of them founded are questionable, if not positively false, and in practice they have proved to be much more hazardous than their supporters would wish us to believe. We should be very loth to meddle much with veins. We seldom do much good, and we may at at any time do harm. The last proposal has been that of Dr. Fricke, of Hamburg. He has treated varicocele, circocele, and varix, by passing a fine thread through the dilated vein, and allowing it to remain from twenty-four to forty-eight hours, according to the degree of increased action excited; and after trying the method in sixty cases, he recommends it as being not more simple in the performance than it is safe and certain in its effects. The simple objection to all operations of the sort is this. They most of them produce some inflammation in the vein. Now phlebitis is of that character, that, once excited, we never can be certain when or where it will be limited. This is an objection founded on the hazard of the operation. There are others, arising from its inefficiency. The argument adduced in support of these operations is a very strong one against them. Phlebitis, it is urged, may follow even venæsection. Exactly so, and if it does succeed so simple a proceeding, how much more likely is it to result from one which is not simple?

10. *Reduction of Dislocations and Dynamometer.*

"Ancient dislocations of the hip-joint have been reduced from forty to nearly an hundred days after the accident; dislocations of the shoulder-joint of very long standing are also reported within the year to have been replaced. Luxations of both radius and ulna backwards have been reduced at sixty-three, and even at seventy days; but it may be well to mention, that in one of these cases the olecranon was fractured in the attempt (owing to *the obstinate resistance of the triceps)*, which a little retarded the cure, as the fracture took five weeks to unite; the patient regained the full use of the limb. The most important contribution in this branch of surgery which it falls within my province to name, is the Memoir of Malgaigne, a document rich in pathological details and novel views, yet so extensive as not to allow of my doing more than mention it in these commendatory terms. This surgeon has repeatedly used the *dynamometer*, for measuring the degree of extension employed with the pulleys; in one case the extending force applied was equal to 250 or 300 pounds. Mr. Weiss, of London, has constructed a *dynamometer* for me, which seems to answer every purpose; I conceive that such an instrument, constantly used when the pulleys are required, may be turned to great practical advantage; it cannot fail to indicate correctly the degree of force applied in each case, and as experience accumulates, serviceable rules, as to the degree of extension admissible at different ages, and on particular joints, may ultimately be laid down in every systematic treatise upon the subject."

11. *Odds and Ends.*

*a.* Mr. Lewis has written on the palliative treatment of hydrocele by acupuncture. We tried this two or three years ago in several cases. We might just as well have read a chapter of Tom Jones over the scrotum; *that,*

indeed would have been a pleasanter proceeding both to the surgeon and the patient, and every whit as effectual.

*b.* Dr. Fricke treats inflammation of the testicle by strapping. If the doctor should chance to get "hernia humoralis" himself, we recommend him to apply the remedy. When we recollect that this disease is usually the result of extension of inflammatory action along the cord, and that it invades the fibrous as well as mucous membranes, we shall see more cause to admire the novelty than to anticipate much from the success of such a measure. It is one of those amusing bubbles that ingenious men occupy themselves in blowing, and other people in endeavouring to catch.

" *c. A small Family.*

In a respectable Italian journal, I find it affirmed, that a lady had forty-five children, (including some abortions,) and often with an interval of only five or six months; and the writer asks whether a double uterus might not have been present, to explain this extraordinary instance of fecundity? Such duplicate organs, we well know, are occasionally met with; in the same journal just referred to, a case of double urinary bladder is described, with the particulars of the dissection."

Well may Mr. Crosse slily hint, that the readers of Journals should possess a large swallow. It is indeed marvellous to look on the rubbish that is periodically printed—so many niaiseries—so many extravagant fancies—and so many downright lies. The profession need a critical journal to keep down the quantity of current trash.

" *d. Extraordinary Delivery.*

Malignant soft tumors, whether of the uterus or ovarium, when they present themselves in the vagina at an advanced period of utero-gestation, give to the less experienced medical attendant the idea of a *placenta prævia*, and many have acted under this erroneous impression. One of the most extraordinary cases I ever was summoned to, proved to be of this description; the operator passed his hand through the soft tumour in the vagina, and, missing the uterus, entered the abdominal cavity, seized and ruptured the gall-bladder, and actually delivered numerous biliary calculi *per vaginam!**"

The concluding portions of Mr. Crosse's elaborate and excellent Address are devoted to the consideration of matters of a less practical, yet not less interesting character. They touch on the state of the medical press, general as well as periodical—on the application of lithography to the elucidation of anatomical or pathological science—on medical statistics—and on other matters of a similar description. Perhaps the most interesting subject to us is, that of medical periodical literature. Yet, though we may feel flattered at the manner in which we are particularly alluded to, we do not suppose that our readers can participate in our feelings on the subject, and we therefore dismiss it with this simple expression of thanks to Mr. Crosse for his good opinion.

But there is a point on which we cannot forbear from offering a remark or two. Mr. Crosse has forcibly pointed out the evils which have sprung from the mode in which the weekly medical press is conducted, and the vices which

* "I communicated this case sometime since to Professor Naeglé, for publication in a foreign journal. I was not summoned until after the decease of the patient; and in the presence of three practitioners I opened the body. Numerous fungoid tumours arose from the right ovarium, and one of them, descending into the pelvis, had presented in the vagina, and being torn, furnished the hæmorrhage; pieces of this soft tumour were removed, and appeared to be the placenta, and some loss of blood continuing, delivery was determined upon, but no fœtus could be found; the hand of the accoucheur had passed through the soft tumour occupying the vagina, into the peritoneal cavity, and the gall-bladder, filled with biliary calculi, had been seized in the search for the foot of the fœtus. Dissection verified the occurrences as I have stated them; the uterus and its appendages are in my pathological collection."

are in some measure inherent in its constitution. It would be an injury to medical literature, if the weekly journals became the sole vehicles of criticism. There are, and probably there ever will be, political parties in the medical profession. Faction is as much the child of liberty, as any of its numerous blessings are, and whilst in a free country there are some who get, and many more who want to get, the two will always be opposed in politics.

Now the weekly journals are peculiarly the organs of political feelings, and it is Utopian to imagine that they ever can be otherwise. All we can reasonably expect from them is, that they will evince some regard to justice and to *truth*. Unfortunately that has not been always the case.

It is hardly possible that a political journal can be a scientific one. Party predilections will insensibly or avowedly influence criticism, and if once suspected, still more if convicted of unfairness, its utility is irretrievably damaged.

Independently of these considerations, criticism of a high order, and circumstantial analysis are incompatible with the constitution of a weekly journal. Rapid in its revolutions, confined in its dimensions, motley in its character, and in some degree ephemeral in interest, it assimilates more to the nature of a newspaper than of a review, and elaborate notices of works are neither possible, nor, if possible, would they be tolerated. Variety is at once the essence and the charm of a weekly journal, and it may even be doubted if the present mania for publishing elementary lectures in its pages is not carried a little too far. Be that as it may, it is certain that its form is not calculated for reviews, and that a journal of greater bulk, of greater gravity of character, of more freedom from political bias and from personal considerations, and with longer intervals between its times of publication, is necessary for analysis and biblical criticism.

On these, and on many other accounts, we think that it would be an evil, and a great one too, if the quarterly medical journals should become extinct. Yet there are those who have declared that the weekly journals are sufficient for the intellectual wants of the profession, and represent its intellectual appetites. On the occasion of the establishment of a new quarterly journal, a short time back, the editor of one of the weekly journals, observed that the Medico-Chirurgical Review was too popular and too well-conducted to render a new periodical necessary, and that, in fact, the quarterly journals were become effete and altogether superfluous. We thank our contemporary for the personal compliment, but must stand forward to defend our order. We admit the ability with which the weekly journals are conducted, and we are sure that they are now indispensably necessary. They are the newspapers of the profession, and it cannot do without them. But, as we have already stated, not only their form, but their very constitution unfits them for elaborate analysis, or for the higher purposes of criticism. When so many works are published, some good, many indifferent, and some utterly worthless, one critical review, at least, is wanted, one quarterly journal, if not more, is indispensable.

It may be urged that analysis and biblical criticism might be consistent with the scope and objects of a weekly journal, supposing that political partialities could be restrained. But that is impossible. There are not only two great political parties in medicine, as in all else, but from the very constitution of things, there ever will be. The two antagonistic principles of inertia and change are daily assuming more distinctness, are daily becoming more opposed, and time which softens most other animosities must necessarily perpetuate, if not aggravate the virulence of the parties in this quarrel. Newspapers in all essential respects, the weekly journals must hold their existence by the tenure of party feelings, and by the advocacy of party interests. Two experiments have been made to determine if a journal can be supported without party, and those experiments have signally failed.

The criticism of medical books differs from that of works in general literature in one important respect. The medical critic must not simply be a learned man, nor a clever man, he must be also a practical man. A practical man, in our profession, is one who sees something, at least, of practice, and who hopes to see more. But such a man will never connect himself with the management of the weekly press, for whichever side he takes, the political animosity that he must encounter will, he knows well, injure his professional prospects. That is another reason, and the most powerful of all, why the biblical criticism of the weekly press can never attain to much respectability.—The radical journal can command only the services of low adventurers, who knowing little, cannot tell us much. The conservative journal cannot, in this instance, obtain the services of those whom it supports, because they are conscious that the advantage is not proportionate to the trouble and the risk.

The only chance which the profession has of obtaining criticism at all worthy of a scientific body, is by its support of the quarterly journals. They are so little mixed up with politics,* that men in good practice, or expecting good practice, do not dread writing in them, and the profession has the advantage of seeing books reviewed by men of education, and of practical attainments.

But we shall say no more at present on this subject, and content ourselves with reiterating the opinion, that the present state of the profession renders the existence of quarterly, as well as of weekly journals, both highly desirable and necessary. We may quote, in conclusion, a note of Mr. Crosse's in which he has presented us with an enumeration of the medical journals published in Europe and America.

"In Great Britain, exclusive of strictly popular works, such as *The Doctor, The Gazette of Health,* &c. we have now *seven* medical periodicals, *three* of them issuing quarterly, *one* two-monthly, and *three* weekly. An ophthalmic journal has just been proposed by Mr. Middlemore; a journal devoted to midwifery and the diseases of women and children, such as has long proved successful in Germany, would, I conceive, embrace a more extensive field, and have a better chance of success; or a separate journal of pharmacy, &c., such as exists in France, Italy, and Germany, and even in America. In the United States of America, as many as eighteen distinct journals upon medical science are enumerated at the present time, issuing at Philadelphia, New York, Boston, Baltimore, Charleston, Cincinnati, and Lexington; and a writer observes that, owing to such channels of information, 'each member of the profession, however remotely located, may now be placed in almost immediate possession of all the important contributions made to our science in every part of the civilized world.' Turning to France, we find nearly *twenty* periodicals upon medicine and its collateral branches; about *fifteen* of these issue from the press at Paris, and are republished in a monthly volume at Brussels, under the title of *Encyclographie des Sciences Médicales.* Belgium has two journals. Seven medical journals, including those of a popular character, were continued in Holland during the last year, the titles of which have been furnished me by Dr. Nieuwenhuys. In Denmark we learn from Dr. Otto that there are three journals; and in Spain there are two. Those published in the Italian language amount to nearly *twenty,* the principal amongst them issuing at Milan, Bologna, Venice, Pavia, Naples, Rome, Turin, Palermo, and Verona. In the German States, and in the German language, *forty-five* journals on the different branches of the medical sciences were actually sent forth in 1835, as I am informed by Dr. Bluff, who has so repeatedly favoured me with information; this is an increase of *five* compared with the preceding year: the demand for so many channels of infor-

* The Lancet, some time ago, lamented that there was not a decidedly radical quarterly journal. It promised such a journal success. In this it displayed a striking ignorance of the principles on which a quarterly periodical can alone expect to exist.

mation is increased by Scandinavian and Russian contributors and readers. After much enquiry under favourable opportunities, I find reason to believe that the extensive territory of Russia has no native medical journal or review, but is supplied from Germany and other countries. The recent increase of medical journalism is indicated in the East Indies, in the West Indies, and in South America."

This Retrospective Address of Mr. Crosse is highly creditable to his good feeling, his good sense, and his research. It will repay any one amply who peruses it.

---

ON TIC DOULOUREUX, OR NEURALGIA, TOGETHER WITH A FEW CURSORY REMARKS ON INTERMITTENT FEVER AND ITS SUPPOSED CONNEXION WITH THE FORMER. By JAMES HEYGATE, M.D. M.R.C.S.L. &c. and Physician to the Derbyshire General Infirmary. Octavo, pp. 64, sewed.

THE author observes in his preface, that—"the fact that severe facial tic douloureux often originates in organic disease of the head, seems to have been scarcely ever entertained." This, we think, is rather premature. Sir Henry Halford relates several cases, where the disease originated in the head—and, among others, that of the late Dr. Pemberton, where, besides unusual thickness of the os frontis, there was found an osseous deposit in the falciform process of the dura mater, with marks of cerebral irritation and inflammation in the neighbourhood. In stating another case, Sir Henry observes:

"The head was opened after death, and an enormous thickening was observed of the frontal, ethmoidal, and sphenoidal bones, in one part to the extent of half an inch; and the anterior lobes of the brain were curiously moulded and indented by the thickened bone. There was thickening also of the whole of the cranium, but not to so great a degree any where as in the parts which have just been named.

Thus we have a demonstration of a *bony deposit proving a cause of pressure on the brain and nerves, and from its situation this must have acted especially on the branches of the fifth pair.*"

Dr. M'Culloch, while tracing tic douloureux to malaria, considers that the cause makes an impression on the whole nervous system (including the brain, of course), though the immediate seat of the pain may be in one particular nerve. Those cases of TIC, which have persevered after the nerve was divided, or even the limb amputated, have been generally referred to the brain.

Our author's attention was particularly attracted to the present subject by a case, which is sketched in the pamphlet under review. He admits that many cases of the disease are dependent on functional derangement of the digestive organs—and that functional disorder in the head can seldom be discriminated from organic disease, till the former has made some progress.

The history and description of the complaint we shall pass over, as being well known to our readers. In respect to the causes, our author thinks that the *simple* cases are generally dependent on disorder of the digestive organs—the *severe*, on "some organic disease," as tumor, ulceration, spicula of bone pressing on the origin of the nerves. "In fact, any substance that presses on the nerves at their origins, or in their course, may certainly, by the irritation it produces, cause neuralgia at the ends of the nerves." A review of the treatment of neuralgia is taken by our author; but we must also pass this over, and come to the case which gave origin to the pamphlet.

Mrs. B. aged 53, had laboured for some time under ophthalmic inflammation, unaccompanied by pain, and reluctantly giving way to the usual remedies. She afterwards became affected with pain in the left side of the head, over the temple. It was slightly mitigated by bleeding, blistering, &c.; but, after some weeks, it assumed the character of "FACIAL TIC DOULOUREUX." At first, the attacks were preceded by a rigor, occurring every evening about six o'clock, and generally lasting till two o'clock in the morning. The pain was chiefly in the course of the supra-orbital nerve. The periodical character, however, was subsequently lost, and pains extended over the cheek-bone to the nose and lip, shewing two branches of the fifth pair to be affected. The pain was very intense, and the line of demarcation between the nerves of the two sides of the face was clearly defined. Numbness extended to the left half of the tongue. Dr. Robertson, of Northampton (one of the most talented physicians in England) was consulted, and assisted with his able advice. The auditory nerve became affected, and the digestive organs deranged. Little benefit was derived from any plan of treatment, and our author began to suspect some organic disease at the origin of the nerves in the brain. Opiates, internally and externally, were the only remedies that mitigated the severity of the pains.

"After suffering for about 4 months in this way, it was perceived that the left eye-ball began by degrees to protrude, till it was pushed out to a very considerable extent. Whilst this was going on, the power of vision in this eye became completely lost, as well as the ability to move it. Then followed at first in a trifling degree, and afterwards more and more profusely, an acrid discharge from the left nostril, of a very offensive nature. It was now clear that there must be ulceration, and, from the nature and seat of the pain, it was reasonable to infer that this ulceration was seated where the nerves supplying the head and face make their exit from the brain. Mrs. B. gradually lost the power of mastication, and her speech became also very indistinct. During this time it was obvious enough that diseased action was extending its ravages;—the glands on each side of the neck became enlarged, and ultimately encroached on the œsophagus; so much so, as to render the passing of fluids only allowable.

After the discharge from the left nostril had continued, and increased in offensiveness, for about three months, bloody matter began to flow from the right nostril also, and the sight of the eye on that side gradually diminished, till at last she could not distinguish any person or object, however closely presented to her view. Thus things went on from bad to worse—the tumours in the neck increasing, the distortion of the features becoming greater, the spasmodic pains, remarkably acute, had reached the right side of the face also; inability to raise herself in bed, and amazing prostration of strength."

"She became extremely emaciated, and after suffering the most excruciating agony, with exemplary patience, and with only short intervals of ease, for upwards of a year, she was seized with a diarrhœa, which had frequently before threatened, and which now terminated a most deplorable state of existence."

Post-mortem Examination.

"On dividing the dura mater, and gradually lifting up the brain, I discovered a tumour (which I have preserved in spirits) at the base, about the size of a pullet's egg, resting partly upon the left crus cerebri and tuber annulare. On cutting into the tumour, it appeared of a hard, white cartilaginous consistency. The surface of the tumour on the fore-part was ulcerated, as were the parts of the ethmoidal and sphenoidal bones adjoining.

The great pressure produced by this extraneous body on the left optic nerve, accounted for the loss of sight in that eye. Ulceration had likewise extended itself along the course of the optic nerve, and matter was found seated behind the eye-ball. This accumulation accounted for the eye-ball being protruded. Ulceration had also extended itself to the right optic nerve, and, from the pressure of the purulent matter which surrounded the nerve, we may account for the sight of that eye, in the latter part of the illness, giving way.

The scalpel, through the ulcerated parts, passed very readily into the nostrils and left eye-ball. There was more water in the ventricles than is usually found. No other deviations from health were observable in the brain. The abdominal viscera were not examined, neither was the structure of the glandular enlargements in the neck. Further dissection was omitted out of delicacy to the feelings of the surviving friends. I do not, however, consider it would have thrown any additional light on the case."

We consider Dr. Heygate's case as the most valuable part of the work, because it adds a very valuable item to the pathology of a most formidable and agonizing disease. Dr. H. corroborates the opinions of a distinguished physician—Sir Henry Halford—and he refers to authors, who are well known in Modern Babylon to be somewhat *apocryphal*. We return thanks, however to the author for this contribution to medical science.

---

The British Medical Almanack, 1837. Edited by William Farr. 12mo, pp. 215. Price, with the Supplement, three Shillings; or bound and gilt, with Writing-paper to record cases, five Shillings.

This little book is a symbol of the times, the active, stirring times we live in. Every class of people has now its appropriate almanack, that is, a calendar useful to all, and the particular information required by itself. There is the commercial almanack, full of money matters—the clerical almanack, which talks of fat prebendaries and golden sees—the comic almanack, for those who like fun—and before us is the medical almanack.

The nature of its contents may be gathered from the following table, which we extract. It treats successively of—

"The Calendar.

A Chronological History of Medicine.

London.—The College of Physicians; the College of Surgeons; Apothecaries' Company; Licentiates and Graduates, 1835-6; Medical and Scientific Societies; Benevolent Societies; Table of Meetings of Societies; Medical Department of the Army; Medical Department of the Navy; a Tabular View of the Medical Schools; Fees; Days and Hours of Lecturing; Hospitals; Table of the Fees; Infirmaries; Dispensaries; Museums; Libraries; Universities.

Provincial Medical and Surgical Association.

Prov. Hospitals; Societies; Schools.

Edinburgh.—University; Colleges; Societies; Infirmary; Dispensaries.

Glasgow.—Medical Institutions.

Aberdeen and St. Andrew's Universities, &c.

Dublin, Cork.—Medical Institutions.

SUPPLEMENT.

The Rise and Progress of the Provincial Medical and Surgical Association.

Statistics of Mortality and Sickness. By T. R. Edmonds, Esq. B.A.

Weight of the Human Body and Brain at all Ages.

Practice of the 14 principal Hospitals in England respecting the Post-mortem Examination of Patients.

Weights and Measures.—Comparison, of Foreign with London Apothecaries' Weight.

Auscultation and Percussion.

Formulary of New Medicines.—(Magendie's abridged, with many additional substances.)

National Statistics.

On the Mortality of Officers in the English Army; the Influence of different Months on the Mortality: by Lieutenant Tulloch.

Description of a Wet Bulb Hygrometer, by J. A. Mason, M.D. (with a Plate).

Statistics of Hospitals.

Table of the British Hospitals.

Acts of Parliament.

Miscellaneous Articles."

Before we say any thing in praise of the Almanack, we must point out a very gross fault in it. If there is any body who has no business with politics, it is surely an almanack-maker. His business is to record facts, not to pester his readers with his political lucubrations. People buy an almanack as an almanack, not as a party-paper; if they find it such, they have great reason to complain of being cheated, and the proprietors may be pretty sure that the aggrieved purchasers will not be duped again. The politics of the almanack may be good or bad—that is not the question. There ought to be no politics in it at all. Radicals are apt to think that there are no such folks as tories, and tories that there are hardly such canaille as radicals. It is very disagreeable for the one party or the other, on opening a calendar which they have unwarily bought, to find a blustering political adversary. That this fault is committed by the almanack-writer before us, will be evident from the passage we proceed to quote. It is contained in the introduction, a dogmatic and rather impertinent preface of three or four pages.

"On passing to the societies, the schools, and the hospitals in the country, it will appear that here also there has been progress. In his eloquent discourses on Medical reform, Mr. Lawrence had to inform the Council of the College of Surgeons that there were *Surgeons* and hospitals in the country; the College of Physicians had connaissance of none but a few gentlemen, whom it stamped as *Extra*—Licentiates in the land of utter darkness, seven miles removed from London. Such was the state of things not many years ago; now turn to the Almanack, of 1837, from Aylesbury, Bristol, and Bath, to York, and count the names of the men as much distinguished by useful labours, or as zealously striving in the cause of science as their most successful brethren in London. The metropolis always derived its strength and talent from the country; its advantages rest on the basis that it is nearer to all the remote parts of the kingdom than they are to each other; it is the centre of the empire, which places every town and province in communication with the rest. But the intelligence and the power of the Medical Profession can no longer be circumscribed by a line drawn seven miles round—the London circle must extend to the shores of these islands."

All this is very true, and our readers know that the passage represents our own sentiments. But it is utterly out of place, and although there are many who entertain these sentiments, there are many, also, who will think themselves snubbed by such remarks. If the publishers regard their own interests, they will alter this hereafter. An almanack is not a journal.

We concur in the following observations, which are, in fact, an echo of our own often-expressed opinions. But there is an unpleasant pertness in their tone, as, indeed, there is in too many passages. They remind us of some observations by Mr. Edmonds himself in the Lancet, which were characterised by a degree of brusquerie and egotism, totally out of place in a paper on statistics.

"The statistics of mortality and sickness form the basis of medical science. Without a knowledge of the laws of mortality and disease on a large scale, and without being well versed in the methods by which they are deduced, the physician practises unguided by the light of general principles, which, even in individual cases, should never be forgotten. The application of the Constant—discovered by Mr. Edmonds in investigating the rate of general mortality—to the sickness and the fatality of attacks at each age, is one of the most important accessions lately made to pathology. Numbers, although they have contributed so much to the progress of other sciences, have been very sparingly applied to medicine. This is partly the fault of the schools. Mathematics were cultivated in the English Universities, but their application to the natural sciences was not pointed out; and the abstract principles escaped from the mind. Mathematics did not form a part of the studies at the Scotch Universities, where a majority of the English Physicians were educated; and in London these principles are still neglected for Latin,—an instrument of insignificant importance when weighed against the elements of arithmetical science. A good authority stated, that there were four members in the *unreformed* House of Commons who, on an emergency, could convert a vulgar into a decimal fraction. The mathematics of our presiding bodies are, we hope, in a more flourishing state. Medical statistics, at present, only require a knowledge of the simplest rules of arithmetic; but the applied mathematics would be an excellent substitute for Latin in the examinations."

The defects in the Almanack can be easily removed. Let the Editor hereafter avoid any political allusions, as he would a tub of aquafortis, and let him shake off that positiveness of manner which is at present disagreeably perceptible. There are some errors of fact. The School of Anatomy of St. George's Hospital is put in Grosvenor Place. The Hospital Prospectus shews that it is in Kinnerton Street.

Of the execution of the remainder of the Almanack we can speak in very favourable terms. The chronological history extends from the year 600 A.D. to 1658. There is much in it to interest and to instruct. The statistical papers are worth reading, and we shall notice them more particularly in our clinical department. We shall conclude this notice by extracting a summary of some observations made by M. Quetelet, in Belgium, on the weight of the human body at different ages. The table, which we need not insert, shews:—

1st. That the weight of the male infant, at birth, is nearly 7lbs. avoirdupois; while that of the female is not quite 6½lbs.

2nd. That the maximum weight (140½lbs.) of the male is attained at the age of 40; while that of the female (nearly 124lbs.) is not attained till 50: from which ages they decline afterwards; the male to 127½lbs. the female to 109lbs.—nearly a stone.

3rd. That the full grown adult is twenty times as heavy as the new-born infant.

4th. That the rate of growth varies: in the first year, the child triples its weight; afterwards the growth proceeds in geometrical progression, so that if fifty infants in their first year weigh 1000lbs., they will in the second weigh 1210lbs.; in the third 1331lbs.; in the fourth 1464lbs.; the rate remaining very constant up to the ages of 11—12 in females; and 12—13 in males, where it must be nearly doubled; afterwards it may be continued, and will be found very nearly correct up to the age of 18 or 19; when the growth proceeds very slowly up to 40. The weight of any number of children between 2 and 9 years of age being known, their weight, the amount of matter they can incorporate in twelve months or two years may be calculated.

Of course such observations are imperfect, and must not be taken for more than they are worth.

We cannot shut the book without strongly recommending it to our readers. It is an useful, an instructive, and an amusing Almanack, and should be on the medical man's library table.

---

### RECENT WORKS ON ANATOMY.

1. THE CYCLOPÆDIA OF ANATOMY AND PHYSIOLOGY. Edited by ROBERT TODD, M.D. Professor of Physiology and General Anatomy in King's College, London, &c. &c. Parts VII. and VIII.
2. THE HUMAN BRAIN, ITS CONFIGURATION, STRUCTURE, DEVELOPMENT, AND PHYSIOLOGY; ILLUSTRATED BY REFERENCES TO THE NERVOUS SYSTEM IN THE LOWER CLASSES OF ANIMALS. By SAMUEL SOLLY, Lecturer on Anatomy and Physiology in St. Thomas's Hospital, &c. With twelve plates, 12mo. pp. 492. London, 1836.
3. A PRACTICAL DEMONSTRATION OF THE HUMAN SKELETON. By GEORGE ELKINGTON, Demonstrator of Anatomy in the Royal School of Medicine and Surgery, Birmingham, &c.
4. ENGRAVINGS OF VARIETIES IN THE ORIGIN, COURSE, AND DISTRIBUTION OF THE ARTERIES; CHIEFLY FROM ORIGINAL OBSERVATIONS AND DRAWINGS, WITH DESCRIPTIVE LETTER PRESS. By ROBERT KNOX, F.R.S.E. &c. &c., and F. J. KNOX, Surgeon, Author of the Anatomist's Instructor, &c. Fasciculus I. One plate.
5. ANATOMICAL ENGRAVINGS. By JONES QUAIN. The Arteries.

It is with feelings of unmixed satisfaction that we see such works as the above on the science of anatomy issue from the press in this country. They exhibit the strong interest awakened in the profession, and the conviction that is entertained of the necessity of implanting an accurate knowledge of anatomical facts in the student's mind.

Two of the works before us are possessed of great merit. The Cyclopædia of Anatomy and Physiology is highly creditable to its authors, and to the public taste. Mr. Solly's book is of a very philosophical character, and it too reflects credit both upon its author and on those who patronise it. The other works are of a different complexion, though all are good in their way.

The pages of a practical medical journal are not adapted for the analysis, nor indeed for the critical review of an elementary science of facts, like anatomy. Yet the advances which have lately been made in it, the light which has been thrown on physiology, and the growing taste for comparative anatomy, render a more extended notice than has hitherto been given by journalists, a tribute equally due to authors and advantageous to their readers. We shall take an opportunity, from time to time, of selecting such anatomical or physiological subjects as we think adapted for the purpose, and presenting a sketch of what is at this moment known of them.

The parts of the Cyclopædia before us commence with the conclusion of the description of the Cilia, and terminate with a paper on Death, by Dr. Symonds. That paper has been noticed in another place. The articles comprised between the first and last are—Circulation, by Dr. Allen Thompson—Cirrhopoda, by Dr. Coldstream—Cirronosis, by Dr. Todd—Conchifera, by M. Deshayes—Contractility, by Dr. Alison—Cranium, by Mr. Malyn—Regions of the Cranium, by Dr. Todd—Crustacea, by Dr. Milne Edwards—Cyst, by Mr. B. Phillips. The variety and importance of the subjects correspond to the high characters of the several authors, and, on the whole, we may very safely say that these parts maintain the high and merited reputation of their predecessors. That would be a poor medical library which wanted the Cyclopædia of Anatomy upon its shelves.

To give clearness to anatomical descriptions, and indeed to render the details of comparative anatomy intelligible, diagrams are absolutely indispensable. The publishers of the Cyclopædia have wisely and liberally illustrated the text with numerous woodcuts. The circulation of the blood in man and other animals is displayed in twenty diagrams, a judicious and economical profusion. One considerable element in the success of the Cyclopædia, is this abundance of drawings, and we would strongly recommend the proprietors to proceed as they have begun, and to spare no expense in the elucidation of minute points of philosophical and comparative anatomy by wood-cuts. Our present crippled space forbids our doing more than add, that, as this work has now arrived at its eighth number, and supported the promise that its early numbers gave, those who at first hesitated to purchase what might never be completed, need entertain no further scruples. It is a work which is peculiarly valuable to the student, because it gives him information that his means would forbid his otherwise obtaining.

The Description of the Human Brain by Mr. Solly is a work, excellent in its conception, and very meritorious in its execution. The time has come when a revolution must take place in the mode of teaching and of learning the anatomy of the brain. The method of slicing that organ to its base, though well enough adapted for the ordinary purposes of pathology, is absurd as a means of becoming acquainted with its healthy structure. That must be learnt by pursuing the fibres of which the cerebro-spinal system is composed, and by accurately studying their relations, and their distribution. When the student has grown conversant with them, a wider and a fairer field is before him. Beginning with the lowest class of animals, he traces, in ascending series, the development of the nervous system, and sees successively additions of new parts and of new functions. Nothing can be more philosophically beautiful than such a study. It amply recompenses him for the fatigue of acquiring the facts which he must do, to enable him to commence such a survey.

" By pursuing this course," says Mr. Solly, in his introduction, " we shall be rewarded by finding that the enkephalon, this apparently most complicated organ in the human being, is but a gradual development from an extremely simple fundamental type on one uniform and harmonious plan, and that the seeming complexity of the cerebro-spinal axis in man really arises from the great concentration, as opposed to the extreme diffusion of its component parts in the lower order of animals; for in no particular are the higher orders more strikingly distinguished from the lower than in the concentration of function within circumscribed spaces."

The plan adopted by Mr. Solly in the arrangement of his matériel is this. He first sketches the comparative anatomy of the nervous system. Commencing with the investigation of the nervous matter, he proceeds to investigate its forms. He views them in the articulata, the mollusca, and the vertebrata. Then he examines, and not in the old fashion, the human brain, the exact anatomy of which he describes. From the brain the transition to the cerebral nerves is natural and proper. The cerebral circulation next engages his attention, after which he treats successively of the development of the brain, of its physiology, and of the physiological inferences which may be drawn from its pathological states.

The execution of the work corresponds with its spirit. It is perspicuous in its style, and lucid in its descriptions. At present we can do no more than commend. At an early opportunity we shall give our readers an opportunity of judging whether our encomia are merited.

Mr. Elkington's practical Demonstration of the Human Skeleton is a very clear, concise, and good system of human osteology. He observes with much truth that there is now no good monograph upon the bones. Monro's is superseded by the descriptions contained in the more exact and elaborate systems of anatomy of Cloquet, Bourgery, &c. A gap then there was, and Mr. Elkington has filled it. We can recommend his book to students, and have no doubt, from its execution, that its author is a demonstrator, worthy of the Birmingham School of Medicine—no inconsiderable compliment.

We have spoken so often of Dr. Quain's Plates, that we hardly know what more to say about them. They are exceedingly well executed, and the only fault we find with them is, that there are not enough of them. We are sure the subscribers would gladly see the work extended, and a sufficient number of plates presented, to constitute a series as complete, at least, as that of Bourgery. This might be done by supplements, and we would strongly recommend it.

The engravings of Varieties in the Arterial System, published under the superintendence of the Knoxes, are deserving of encouragement. Anatomists, like other people, are too apt to become routinists, and to go on at a jog-trot pace, in the customary path. Yet it is of great importance, in a practical sense, to be well acquainted with the varieties in the origin and course of vessels, so peculiarly subjected to operations as the arteries. The present fasciculus displays the following arterial vagaries.

1. The external carotid artery proceeds *anterior*, instead of posterior, to the dygastric and stylo-hyoid muscles.

2. The axillary artery gives off an anomalous branch, which follows the course of the humeral artery, and at the elbow-joint is joined by another anomalous artery *from* the humeral. The vessel that results pursues the course of the radial, and is again reinforced by a large branch near the wrist.

3. The fibular artery nearly equals in size the posterior tibial, with which it coalesces between the internal malleolus and heel.

4. In the same limb, the posterior fibular branch was very small, and the anterior peroneal was an offset from the anterior tibial.

The Knoxes are ever anxious to enrich anatomical science, and we trust their efforts will enrich themselves. We shall notice future fasciculi, when they appear, and we can speak very favourably of this one.

